# Overeaters Anonymous: A Mutual-Help Fellowship for Food Addiction Recovery

**DOI:** 10.3389/fpsyg.2018.01491

**Published:** 2018-08-20

**Authors:** Boris C. Rodríguez-Martín, Belén Gallego-Arjiz

**Affiliations:** Fundación Recal, Madrid, Spain

**Keywords:** Overeaters Anonymous, perceived food addiction, mutual-help groups, addiction recovery, spirituality

The notion that overeating can be an addictive behavior has been accepted by many individuals who suffer from this problem. Overeaters Anonymous (OA) is a 12-step mutual help group founded in 1960 to support individuals who perceive themselves as food addicts (Suler and Barthelomew, 1986).

However, the concept of food addiction is highly controversial (Meule, 2015). Furthermore, there is no consensus regarding whether addictive-like eating should be conceptualized as a substance-related (Schulte et al., 2015) or a behavioral addiction (Hebebrand et al., 2014). Because of this, the core concept of this article is *perceived food addiction* (PFA) rather than an objective and clinically accepted definition of food addiction.

OA members share an abnormal relationship with food. They also share numerous failed attempts to overcome this problem (Russell-Mayhew et al., 2010). Moreover, overeating, restraint, fasting and purging are perceived as both chronic compulsions (despite their negative consequences) and relapsing disorders^1^.

Therefore, eating disorders may be common amongst OA members (von Ranson et al., 2011). Pharmacological interventions, cognitive-behavioral psychotherapy (CBT), interpersonal and other non-specific supportive therapies are the most recommended treatments for individuals with eating disorders (Linardon et al., 2017).

OA has applied the addictive-like eating framework for decades to help members achieve recovery. According to the 12-step program any eating-related problem is regarded as a physical, emotional, and spiritual disorder (Hertz et al., 2012). OA also recommends a set of tools in order to overcome PFA (Russell-Mayhew et al., 2010). Finally, it has been observed that attendance at meetings and the use of other tools in individuals with addictive-like eating behaviors show some benefits (Malenbaum et al., 1988; Westphal and Smith, 1996; Kriz, 2002), but studies with regard to their effectiveness remain scarce (Schulte et al., 2015).

The current article aims to examine the core concepts of OA fellowship and its implication in clinical practice to support an ongoing recovery from perceived food addiction.

## Addiction recovery

According to White (2007) addiction recovery is the experience through which individuals use both internal and external resources to solve problems inflicted by their addiction. This statement includes, managing their continued vulnerability to such problems, and developing a healthy, productive and meaningful life.

## Recovery tools

An interesting qualitative analysis regarding how OA helps its members divided OA tools into two main categories (Russell-Mayhew et al., 2010): *explicit* and *implicit*. The distinction was made in order to differentiate tools described in OA literature (explicit) from those which are less obvious and that are consistently put into practice by fellow members (implicit).

**Figure 1:**
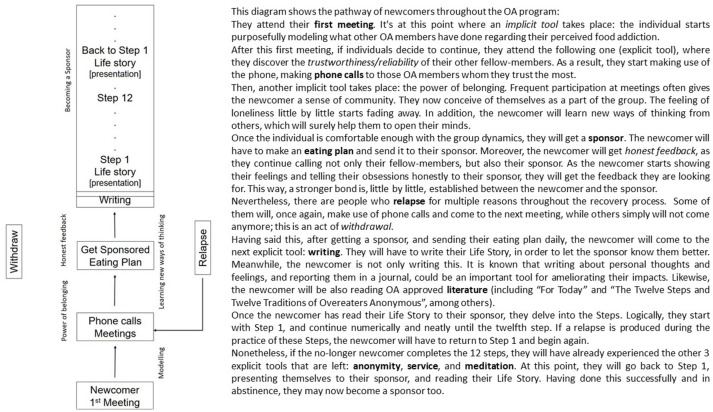
OA recovery pathway diagram: steps and tools.

Explicit tools are those described in the OA approved literature: (1) Plan of Eating; (2) Sponsorship; (3) Meetings; (4) Telephone calls amongst members; (5) Literature revision; (6) Writing about thoughts and feelings (step work); (7) Anonymity, (8) Service and (9) Meditation. On the other hand, implicit tools involve *modeling, learning new ways of thinking, honest feedback*, and *the power of belonging*. Figure 1 displays an OA recovery diagram.

## Defining abstinence from the OA perspective

Abstinence could be broadly defined as the action of refraining from addictive-like eating behaviors. An individual is considered abstinent when he/she is eating food prescribed by a pre-designated food plan at specified meal times during the day (Malenbaum et al., 1988).

Abstinence is further defined as following a food plan that eliminates binge-triggering foods. However, there are critics regarding “forbidden foods” because it could be harmful in the context of addictive-like eating problems (Bermis, 1985; Schulte et al., 2015).

It has been observed that greater adherence to a food plan, higher frequency of phone calls to other members and more time spent writing about one's thoughts and feelings were significant predictors of decreasing relapse frequency. This observation is taken from 231 OA members with binge eating disorder and bulimia nervosa (Kriz, 2002).

## Dealing with food cravings and body/weight concerns

Food cravings are directly related to the failure of suppressing excessive food intake among compulsive overeaters (Rodríguez-Martín and Meule, 2015). In addition, food and body weight/shape related thoughts may play an important role in the maintenance of unhealthy eating behaviors (Rodríguez-Martín et al., 2015). Thus, dealing with both intrusive thoughts and cravings seem essential to keep abstinent in recovery.

The Elaborated Intrusion Theory of Desires^2^ offers a framework that made a distinction between intrusive thoughts, and their elaboration (Kavanagh et al., 2005; May et al., 2012). This theory has been used in clinical settings (May et al., 2014) to prevent the elaboration of food-thoughts in overweight individuals (Rodríguez-Martín et al., 2013a) as well as to support healthy eating in women with anorexia nervosa (Cardi et al., 2012).

Outreaching OA tools such as phone calls to other members and the time spent writing, reading or meditating about personal thoughts and feelings might prevent the elaboration of intrusive thoughts which could lead to relapse. Thought control strategies such as social control and reappraisal (Fehm and Hoyer, 2004) might explain the effectiveness of phone calls to other fellow-members. On the other hand, a simple writing about personal values such as relationships or religious beliefs could be considered an important self-affirmation technique in order to allow women to handle weight-related stress (Logel and Cohen, 2012).

Moreover, when it comes to writing about personal thoughts and feelings, it is demonstrated that this could be an important tool for ameliorating their impacts. Mechanisms of *writing* may be similar to other cognitive-behavioral techniques such Association Splitting (Moritz and Jelinek, 2011). The exercise of writing has been proven to be useful to reduce weight and shape concerns (Musiat et al., 2014) as well as other everyday-life “obsessions” (Rodríguez-Martín et al., 2013b).

## Meetings and social support

Meetings and social support are considered some very important features in 12-steps mutual-help groups (Yates, 2014). Meetings provide a safe ground and an access to positive attachment figures. Both meetings and social support facilitate a corrective emotional experience (Hertz et al., 2012).

Social support has been pointed out as an important element of success for OA members who perceive the program as effective (Russell-Mayhew et al., 2010). From the point of view of the Cultural-Historical approach, the community itself is a collective teacher. Members of the community serve to model a healthy lifestyle to the newcomers (Radionova, 2017). Finally, recovery experience goes through a social identity transition passing from “being an addict” to “being an addict in recovery” (Best et al., 2016).

An interesting study assessed members of OA who attended meetings during an average of five years in OA (Westphal and Smith, 1996). The majority of participants appeared to have met diagnostic criteria for eating disorders in the past, but only 8.8% remained diagnosable at the time of the study.

## Sponsorship

A *sponsor* is a member with significant abstinence time and experience in working all the 12 steps. The sponsor is someone who freely shares his or her experiences and offers guidance on how the newcomer may work the steps (Dossett, 2013). The sponsor is the primary source of emotional support and psychological advice in OA (Suler and Barthelomew, 1986).

A mean of 3 years of abstinence has been observed amongst individuals with bulimia nervosa who attended five OA meetings per week and called their sponsor daily (Malenbaum et al., 1988). Furthermore, having a mentor significantly improves treatment compliance for individuals in recovery from eating disorders (Perez et al., 2014).

## Emotional and spiritual recovery

Emotional and spiritual recovery is also achieved in the context of interpersonal relations developed with the sponsor and other members by following the 12-step program and its tools (Hertz et al., 2012). In fact, many members attribute the effectiveness of the program to emotional and spiritual aspects of recovery (Russell-Mayhew et al., 2010).

A core concept commonly pointed out as a spiritual approach to addiction is powerlessness. Thus, addicts in recovery seek to be given the will to remain abstinent by a Higher Power (Dossett, 2013). The importance of spirituality was also highlighted as predictor of success in members who have been in OA an average of 5 years (Westphal and Smith, 1996).

Nevertheless, there are many sources for spiritual connection for non-religious or skeptical individuals (Hyland et al., 2010): e.g., people, nature, places and the Universe. Hyland et al. (2010) have defined spirituality as a human disposition to experience a special sense of connection with something that is either part of or not part of the natural world which: (1) is experienced as transcending the kind of connection that normally characterizes a person's life and (2) depends on the person's belief systems. We have met individuals with addictive disorders who are skeptical and have chosen the “force of the group” or “the 12-step program” as a personal “construction” of a Higher Power which “works for them” (Dossett, 2013).

A recent qualitative systematic revision of 53 peer-review articles pointed out some spiritual strengths related with long-term addiction recovery (Selvam, 2015): perspective, equanimity, humility, forgiveness, kindness, love and hope. It might be possible to develop such spiritual strengths by systematically using both, *explicit* and *implicit* tools of the Program (Russell-Mayhew et al., 2010).

## Clinical applications

In 2018 a textbook entitled *Processed Food Addiction* which dedicated an entire section to *recovery* was published (Ifland et al., 2018). Concepts such as abstinence, food plans, ability to avoid triggering, prayer and meditation, service and so on, are very similar to the tools offered in OA. Explicit and implicit tools should be studied more deeply in OA. To conduct studies on these tools is especially difficult because of the program's anonymous structure (Russell-Mayhew et al., 2010). OA meetings only allow access to those who identify themselves as food addicts and they do not disclose their identity. For this reason, it could be difficult for researchers to contact the sample.

However, the idea of integrating the 12-steps framework with traditional psychotherapy to treat eating disorders is not new (Johnson and Taylor, 1996). In spite of this, the effectiveness of some therapeutic approaches such as Self-help and its combination with supportive groups such as OA remains largely untested (von Ranson and Farstad, 2014).

A recent systematic revision showed that some non-specific supportive therapies (such as self-help manuals, supportive orientation and supportive expression) were equally efficacious to CBT for eating disorders (Linardon et al., 2017). These therapies usually make use of psychological techniques that are common to all approaches (e.g., providing empathy, discussion of experiences and emotions).

Between 1994 and 1999, in Israel, 409 obese women and 169 with bulimia nervosa were treated in small group settings using the Minnesota twelve-step treatment model (Trotzky, 2002). The majority of women with bulimia nervosa (*n* = 121; 71%) stopped purging behaviors for a minimum of a 6-month period. A mean weight loss of 9.3 Kg was reported for obese women during the same period.

Perhaps, a meta-recovery framework (Winship, 2016) is needed in order to handle addictive-like eating behaviors. Because of this, combining medical assistance with 12-step programs could provide long-term support to individuals after the initial treatment; thus, reducing the costs of mental health services during follow-up care (Galanter, 2017).

## Take-home message

In OA, PFA is regarded as a physical, cognitive, emotional and spiritual disorder. Abstinence is a way of avoiding such addictive-like-eating behaviors, by following a pre-designated food plan.

OA tools such as meetings, food plans, telephone calls and writing tend to be perceived by its members as the most useful to them.

Spirituality is considered as a significant predictor of abstinence success and increased wellbeing.

Combining medical assistance and psychological interventions with the OA framework could provide long-term support to individuals with addictive-like eating behaviors.

Further studies are needed in order to determine OA effectiveness.

## Author contributions

BR-M wrote the initial draft and conceptualized the fundamentals of this manuscript. BG-A reviewed the manuscript several times and substantially contributed to discussions and clarifications around both the topic discussed and its final version.

### Conflict of interest statement

The authors declare that the research was conducted in the absence of any commercial or financial relationships that could be construed as a potential conflict of interest.

## Footnotes

^1^Decades later other 12-step mutual help groups have been founded focused solely on food addiction (Food Addicts Anonymous; 1980s) or unhealthy eating practices (Anorexic and Bulimic Anonymous; 1990s).

^2^In the Elaborated Intrusion Theory of Desires an intrusive thought is the beginning of an elaborative episode for the individual. An intrusive thought could emerge from an associative process linked with: physiological deficit, negative affect, external cues, other cognitive activity and anticipatory responses to the target; but their progressive elaboration is the key process for the cravings' maintenance. Elaborative and associative processes feed among themselves when the target elicits powerful affective reactions or a keen sense of deficit.
